# A single-center observational study on the efficacy of percutaneous coronary intervention for ischemic heart failure: A cohort study

**DOI:** 10.1097/MD.0000000000010238

**Published:** 2018-03-30

**Authors:** Zongtao Wang, Lijun Jin, Wanxing Zhou, Da Lei, Hong Yan, Huimin Yu, Zhihuan Zeng, Guiping Zhu, Jianyi Zheng, Yining Dai, Haifang Wang

**Affiliations:** a The First Affiliated Hospital of Guangdong Pharmaceutical University, School of Clinical Medicine of Guangdong Pharmaceutical University; b Guangdong Cardiovascular Institute, Guangdong General Hospital, Guangdong Academy of Medical Science; c Nanfang Hospital, Southern Medical University, Guangzhou, China.

**Keywords:** cardiac function, cohort, ischemic heart failure, percutaneous coronary intervention, survival

## Abstract

The effects of revascularization by percutaneous coronary intervention (PCI) on cardiac function and clinical outcomes in patients with confirmed coronary artery disease (CAD) and heart failure (HF), on the basis of the optimal medical treatment recommended by current guidelines, remain to be determined.

A cohort study was performed to evaluate the efficacy of PCI on the basis of optimal medical treatment in patients with CAD and HF. Patients who received PCI were subsequently grouped according to partial and complete revascularization (CR) depending on the PCI outcome. The primary outcome was defined as a composite outcome of major adverse cardiovascular events (MACEs). Changes in left ventricular ejection fraction (LVEF) also were compared.

A total of 69 patients (12 who received medical treatment and 57 who received PCI) were included. Patients in the PCI group showed significantly improved LVEF (*P <* .001), but patients in the medical treatment group did not (*P > *.05) after 3 months of follow-up. MACEs occurred in 50% patients in the medical treatment group and 19.3% patients of the PCI group, with this difference almost reaching statistical significance (*P* = .06). Compared with patients who received medical therapy only, patients who received PCI experienced better survival (*P* = .02). Moreover, survival seemed to be better in patients who achieved CR with PCI of the coronary arteries than in those who had partial revascularization of the coronary arteries (*P* = .06).

PCI may be effective for improving survival in patients with CAD and HF.

## 1 Introduction

Despite significant improvements in medication and device-based treatments in recent decades, heart failure (HF) remains one of the most important causes of morbidity and mortality worldwide.^[1–3]^ Ischemic heart disease (IHD), including coronary artery disease (CAD), has been indicated as the most common cause of HF.^[4]^ In China, more than half of HF cases were found to be complicated by CAD.^[5]^ Indeed, partial or complete obstruction of the coronary artery was suggested to lead to apoptosis and necrosis of cardiomyocytes in the ischemic zone of the myocardium, which may be the most important mechanism underlying cardiac systolic dysfunction associated with CAD.^[6]^ Moreover, the cardiomyocytes in the borderline zone of the ischemic myocardium may suffer from stunning or hibernation, which has also been considered as an alternative mechanism underlying the pathogenesis of ischemic cardiac dysfunction.^[7]^ With the improvement of medical skills for the management of acute coronary events, many patients survive conditions such as acute coronary syndrome (ACS), and these patients have been suggested to be at higher risk for development of HF. Therefore, the development of effective treatment strategies for patients with IHD is of important clinical significance for improving the prognosis of patients with CAD and subsequent HF.

Administration of optimal medical treatments for patients with HF, including medications such as beta-adrenergic receptor blockers,^[8]^ angiotensin converting enzyme inhibitors (ACEIs) or angiotensin II receptor blockers (ARBs),^[9]^ and aldosterone receptor antagonists,^[10]^ have been proven to be associated with significantly improved prognosis in patients with HF. These treatments also are recommended for patients with CAD and HF by current major clinical guidelines for HF treatment.^[11–13]^ However, whether revascularization can improve clinical outcomes in patients with CAD and HF remains to be determined.^[14,15]^ Currently, strategies for myocardial revascularization include coronary arterial bypass graft (CABG)^[16]^ and percutaneous coronary intervention (PCI).^[17]^ A recently published large-scale randomized controlled trial (RCT, surgical treatment for ischemic heart failure, STICH trial) including 1212 patients (median follow-up, 9.8 years) with an ejection fraction <35% and CAD amenable to CABG found that CABG on the basis of optimal medical treatment for HF was associated with significantly improved all-cause mortality and the combination of all-cause mortality and cardiovascular hospitalization.^[18]^ Moreover, these clinical benefits of CABG seemed to be more remarkable in younger patients with IHD than in older patients.^[19]^ The results of the STICH study highlight the possibility that achievement of revascularization with the less invasive strategy, PCI, may also be associated with improved clinical outcomes in patients with CAD and HF. However, to the best of our knowledge, few RCTs have been published regarding the clinical benefits of PCI in patients with CAD and HF. Therefore, in this study, we explored the potential effects of PCI as an add-on therapy with optimal medical treatment in patients with CAD and HF, focusing on outcomes of cardiac systolic function and clinical outcomes.

## 2 Methods

This study was a single-center prospective cohort study designed to evaluate the efficacy of PCI based on optimal medical treatment in patients with confirmed CAD and HF. Patient enrollment was performed from October 1, 2013 to June 11, 2014 in the Department of Cardiology of Guangdong General Hospital. The study protocol was approved by the local ethics committee before performance of the study, and all included patients provided written consent before enrollment.

### 2.1 Inclusion and exclusion criteria

Hospitalized patients were included in our study if they met all of the following criteria:

Adult age but <85 years

Had symptoms of angina pectoris for at least 6 months

Had confirmed CAD with ≥50% stenosis of at least 1 major coronary artery on coronary angiography (CAG) or coronary computed tomography angiography (CCTA)

Had symptoms of cardiac dysfunction as evaluated by the New York Heart Association (NYHA) functional class III or IV

Had echocardiographic examination-confirmed cardiac systolic dysfunction of left ventricular ejection fraction (LVEF) of <50%.

Patients with the following conditions were excluded.

Acute myocardial infarction diagnosed within 1 month of enrolment

Documented valvular heart diseases or congenital heart diseases for which a surgical or an interventional repair may be needed or malignant diseases (e.g., cancer) and those with a life expectancy of <3 months.

### 2.2 Medical interventions and study protocols

Baseline evaluation of the patients was performed at admission and included physical examination, biochemical assessment of blood samples, electrocardiographic (ECG) examination, and echocardiographic measurements. All of the included patients were treated with optimal medical treatment according to the recommendations of the American Heart Association (AHA) guidelines for CAD^[20]^ and HF.^[13]^ Moreover, based on the previous findings of CAG or CCTA, some of the patients also underwent PCI therapy based on the judgments of an experienced attending cardiologist following the indications and recommendations of the AHA PCI guidelines.^[21]^ The PCI process was performed by an experienced interventional cardiologist, and the periprocedural treatment of the patients who underwent PCI was based on the recommendations of the above guidelines. The complexity of lesions was categorized according to 3 levels, mild, moderate, and complicated, according the SYNTAX score.^[22]^ The patients who received PCI were further assigned to the complete revascularization (CR) group and partial revascularization (PR) group, depending on whether all of the severe stenosis of the coronary artery was treated during the PCI process. We defined CR as residual stenosis <10%.

### 2.3 Follow-up and outcomes

Patients were scheduled for a follow-up at 3 months from the date of enrollment. The follow-up was performed by an experienced cardiologist in the cardiovascular department, which included briefly obtaining the clinical manifestations and medical histories, serum biomedical assessment, ECG, and another echocardiographic examination. The primary outcome of the study was the change in cardiac systolic function as indicated by the LVEF via ECG. Moreover, the composite clinical outcome of major adverse cardiovascular events (MACEs) in the follow-up was also evaluated. The MACEs included all-cause death and cardiovascular death, nonfatal myocardial infarction, severe HF, recurrence of angina pectoris, rehospitalization due to the aforementioned reasons, and revascularization (CABG or coronary artery stenting).

### 2.4 Statistical analysis

Statistical analyses were performed using SPSS 19.0 software. Continuous variables are presented as means ± standard deviations (SDs), and categorical variables are represented as frequencies and percentages. Comparisons of continuous variables were performed with t-test between 2 groups and analysis of variance (ANOVA) among multiple groups. As for comparisons of categorical variables, the chi-square test or Fisher exact test was applied. Event-rate estimates and clinical benefits were calculated using the Kaplan–Meier method and statistically compared using the log-rank test. A statistically significant difference was considered if *P* < .05, and all statistical tests were 2-tailed.

## 3 Results

### 3.1 Baseline characteristics

Overall, 69 patients with confirmed IHD and HF were included in this study and followed for up to 3 months, and PCI was performed in 57 of these patients. The baseline characteristics of the patients according to whether PCI was performed are presented in Table 1. The baseline characteristics were generally matched between the 2 groups, except that patients in the PCI group appeared to have a better preserved LVEF, although the difference was not quite significant (*P* = .06). The baseline characteristics of patients who experienced CR or PR of the coronary artery are presented in Table 2. Patients with CR had a greater left ventricular end-diastolic diameter (LVDd) than those with PR (*P* = .03), while the other baseline characteristics were generally matched.

**Table 1:**
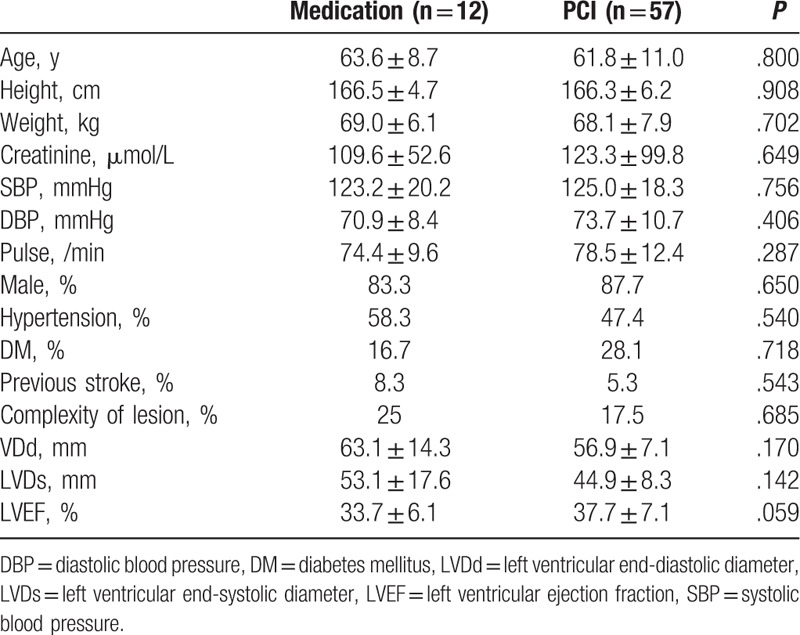
Baseline characteristics of the patients included in the medication and PCI groups.

**Table 2:**
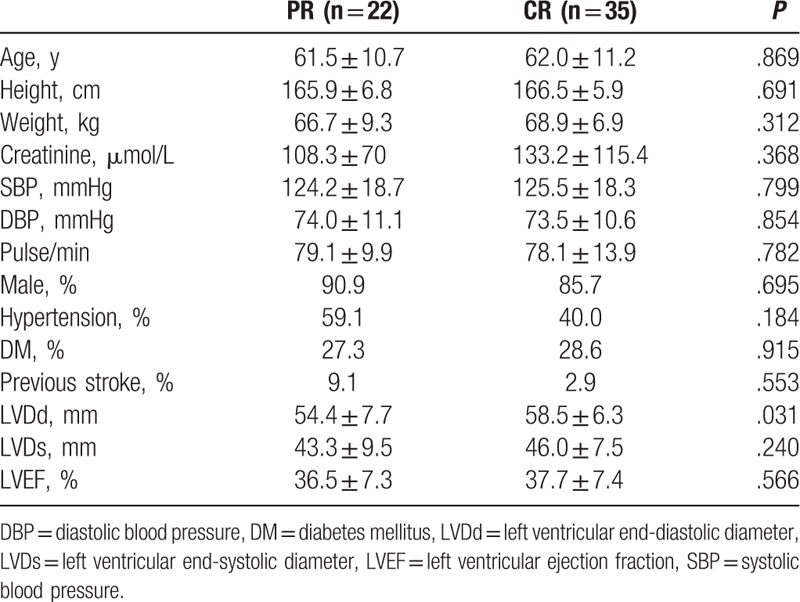
Baseline characteristics of the patients included in the PR and CR groups.

### 3.2 Effects of PCI on cardiac systolic function in patients with CAD and HF

The changes in echocardiographic parameters in patients with CAD and HF following treatment with optimal medication treatment with or without PCI are presented in Table 3. LVDd and left ventricular end-systolic diameter (LVDs) were not significantly affected after treatment for 3 months in each group, and these parameters did not significantly differ between the 2 groups at 3 months after enrollment (*P > *.05). Interestingly, although LVEF was not significantly improved after 3 months of optimal medical treatment, LVEF was significantly improved in the PCI group (*P <* .001). Consistently, the LVEF at 3 months after enrollment was significantly larger in patients in the PCI group than in those in the medical treatment group (38.5 ± 13.9% vs 48.1% ± 11.2%, *P = *.045). These results suggested that PCI may support greater improvement in cardiac systolic function compared with optimal medical treatment only in patients with CAD and HF.

**Table 3:**
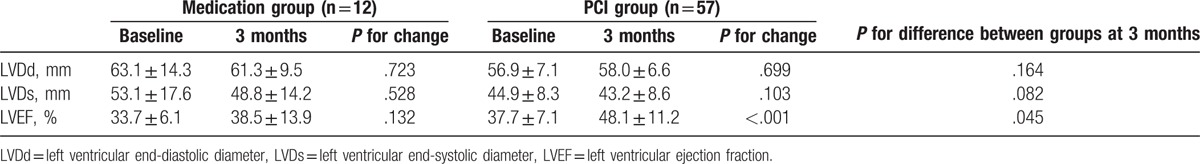
Changes in echocardiographic parameters following PCI.

### 3.3 Effects of PCI on clinical outcomes in patients with CAD and HF

**Figure 1:**
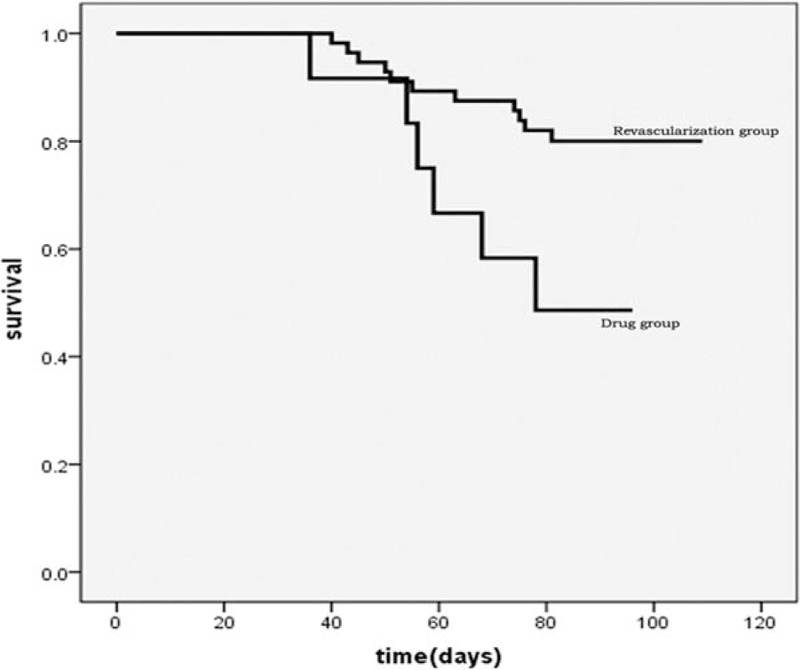
The Kaplan–Meier curves for the survival of patients in the medical treatment and PCI groups within 3 months of follow-up.

**Figure 2:**
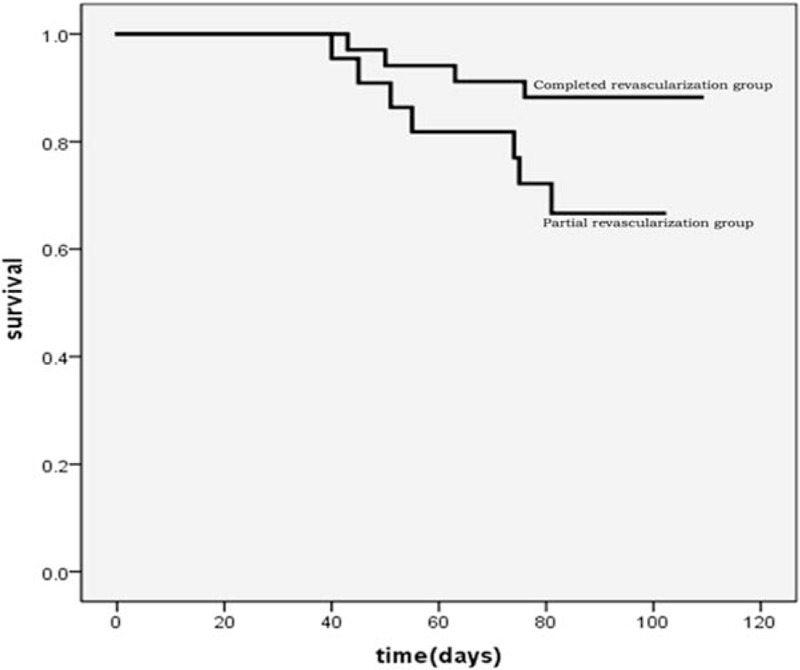
The Kaplan–Meier curves for the survival of patients who experienced complete or partial revascularization of the coronary arteries within 3 months of follow-up.

During the follow-up of 3 months, MACEs occurred in 50% patients in the medical treatment group and 19.3% patients in the PCI group, and this difference was almost significant (*P = *.06). The results of subsequent survival analyses based on Kaplan–Meier plots are presented in Figures 1 and 2. Compared with patients who received medical therapy only, patients who received PCI showed better survival within 3 months (*P = *.02, Fig. 1). Moreover, survival seemed to be better in patients who experienced CR with the PCI of the coronary arteries compared with those who experienced PR of the coronary arteries, with the difference being almost statistically significant (*P = *.06, Fig. 2). The comparison of echocardiographic parameters between PR and CR cases is described in Table 4. The LVDd (56.4 ± 7.7 vs 57.7 ± 7.5, *P = *.023) and LVEF (36.5 ± 7.3% vs 48.6 ± 12.4%, *P = *.007) were significantly affected in the PR group. In the CR group, the LVDd was not significantly affected after treatment for 3 months, whereas the left ventricular end-systolic diameter (LVDs) and LVEF changed significantly after 3 months (LVDs: 46.0 ± 7.5 vs 43.7 ± 8.1, *P = *.012 and LVEF: 37.7 ± 7.4% vs 44.7 ± 10.6%, *P <* .001). However, there were no differences in the LVDd, LVDs, and LVEF between the PR and CR groups. Based on these results, it is unclear whether CR was associated with increased LVEF.

**Table 4:**
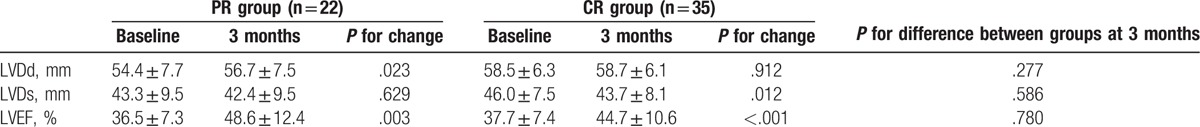
Variation in the PR and CR groups.

## 4 Discussion

In this study, we found that application of PCI in patients with CAD and HF was associated with significantly improved cardiac systolic function within 3 months of follow-up as compared with optimal medical treatment alone. All the lesions of patients were moderate or complex. There was no significant difference in the complexity of lesions treated by PCI between the 2 groups. Moreover, achievement of revascularization by PCI was associated with additional improvement in clinical outcomes as evidenced by the significantly improved survival rate on the basis of optimal medical treatment. Interestingly, the results of subsequent analyses suggested that CR of the coronary artery via PCI did not raise the LVEF for CAD patients with HF, but it seemed to be related t better improved survival outcomes in patients with CAD and HF as compared with PR. These results highlight the possible role of additional clinical benefits of PCI on the basis of optimal medical therapy for patients with CAD and HF. Further RCTs are needed to confirm our results.

Since the recently published STICH trial indicated the potential benefits of CABG for clinical outcomes in patients with CAD and HF on the basis of optimal medical treatment, whether revascularization achieved by PCI is associated with similar additional benefits for cardiac function and survival in these patients again became an important research topic in the treatment of IHF. However, to the best of our knowledge, no RCTs have been published to date regarding the role of PCI in CAD patients with HF. Therefore, the results of prospective cohort studies on the topic may provide some evidence and rationale for RCTs regarding the role of PCI in IHF. Our study found that PCI on the basis of optimal medical treatment was associated with significantly improved cardiac systolic function as compared with optimal treatment alone. These results were consistent with the findings of some previous studies. A recently published retrospective cohort study of 2,229 consecutive HF patients with reduced LVEF found that revascularization by PCI was a significant determinant of LVEF improvement.^[23]^ The mechanism underlying the benefits of PCI for additional improvement in LVEF in IHF patients may be related to the reperfusion of the hibernating myocardium, and this is further supported by the results of a recent study based on cardiac magnetic resonance imaging (CMRI).^[24]^ This study included 29 patients with HF with reduced LVEF and CMRI evidence of viability and/or ischemia within the region supplied by the targeted artery and found that application of PCI was associated with improved cardiac function, reduced serum B-type natriuretic peptide, and clinical prognosis.^[24]^ Since improvement in LVEF has been proven to be a significant predictor of improvement in clinical outcome in patients with HF,^[25]^ these benefits of PCI for cardiac function may be associated with the potential benefits for clinical outcomes such as survival in these patients. Indeed, our study found that PCI based on optimal medical treatment was associated with improved survival within a 3-month follow-up period in patients with CAD and HF, and CR achieved by PCI seemed to confer more remarkable benefits on survival as compared with PR. These results were consistent with the previous findings from a retrospective cohort study of patients with non-ST segment elevated ACS in which revascularization via PCI significantly improved the prognosis of patients presenting with HF, but not of those without HF.^[26]^ Interestingly, a previous meta-analysis including 19 clinical studies found that although achievement of revascularization via PCI in patients with left ventricular systolic dysfunction led to similar clinical outcomes of hospitalization and mortality compared with CABG, neither PCI or CABG improved clinical outcomes beyond pharmacological therapy alone.^[27]^ Of note, most of the included studies in this meta-analysis were published before 2011, and improvements in devices and skills related to PCI- and CABG-mediated revascularization may be the potential reasons for the differing results between the previous meta-analysis and our study and the STICH trial.

Our study has limitations that should be considered when interpreting the results. First, our study was a pilot prospective cohort study with a limited sample size, and the results may be confounded by some characteristics of the patients and interventions. Therefore, the results of our study should be confirmed by studies with larger sample sizes. Although the important baseline characteristics of the patients in the PCI group and the medical group were generally balanced, we cannot exclude the possibility that some other potential variables may not have been matched and subsequently affected the outcome of the study. For example, patients who underwent PCI may have had better cardiac function at baseline, with an almost significant difference detected. This imbalance may have contributed to the favorable outcomes in patients assigned to the PCI group. Thus, RCTs are needed to confirm our results. Secondly, the numbers of patients included in our study were relatively small. Some differences in outcomes, such as the components of the MACE outcome, may become significant if a larger sample size could be achieved for the study. Thirdly, decisions regarding the performance of PCI were made based on the decisions and judgments of the interventional cardiologists based on the results of angiography and CTA according to the PCI guidelines. These processes seemed to be somewhat subjective, and evaluation of myocardial viability was not performed prior to the PCI process. A test of myocardial ischemia and viability could provide guidance for the performance of PCI in these patients to achieve better functional and clinical outcomes. However, these tests were not performed in our study. In view of the fact that improvement of the perfusion of the hibernating myocardium is considered the most important mechanism underlying revascularization in patients with CAD and HF, future research should apply a myocardial viability assessment as guidance for performing the PCI process in these patients.^[28]^ Fourthly, since CABG has been proven to be effective for improving clinical outcomes in patients with CAD and HF, comparisons of clinical outcomes achieved with PCI and CABG in patients with CAD and HF already on the optimal medical treatment are needed in future studies. Finally, also due to the small size of the patient population, whether the PCI process provided additional benefits in particular subgroups of patients was difficult to evaluate. This is important for the identification of patients who may benefit from PCI, since the STICH study found that the clinical benefits of revascularization achieved by CABG may be more remarkable in younger patients with CAD and HF than in elderly patients.^[19]^

## 5 Conclusion

PCI-mediated revascularization may be effective for improving survival, but it did not improve the cardiac systolic function in patients with CAD and HF. Large-scale RCTs are warranted to confirm these findings and evaluate the role of myocardial viability assessment in patients with IHF by PCI-guided revascularization.

## Author contributions

**Conceptualization:** L. Jin, Z. Wang.

**Data curation:** L. Jin, W. Zhou, Z. Wang.

**Formal analysis:** D. Lei, J. Zheng, L. Jin, W. Zhou, Z. Wang.

**Funding acquisition:** L. Jin, Z. Wang.

**Investigation:** D. Lei, H. Wang, H. Yan, H. Yu, L. Jin, Y. Dai, Z. Wang.

**Methodology:** H. Yan, L. Jin, Z. Wang.

**Project administration:** L. Jin, Z. Wang.

**Resources:** H. Yu, L. Jin, Z. Wang.

**Software:** L. Jin, Z. Wang, Z. Zeng.

**Supervision:** G. Zhu, L. Jin, Z. Wang.

**Validation:** L. Jin, Z. Wang.

**Visualization:** L. Jin, Z. Wang.

**Writing – original draft:** L. Jin, Z. Wang.

**Writing – review & editing:** L. Jin, Z. Wang.
